# Desmoplastic Fibroma Recurrence Associated with Tuberous Sclerosis in a Young Patient

**DOI:** 10.1155/2018/1370184

**Published:** 2018-04-18

**Authors:** A. M. Espinoza-Coronado, J. P. Loyola-Rodríguez, J. H. Olvera-Delgado, J. O. García-Cortes, J. F. Reyes-Macías

**Affiliations:** ^1^Advanced Education in General Dentistry, Master Degree Program at San Luis Potosi University, Faculty of Dentistry of San Luis Potosi, Universidad Autónoma de San Luis Potosí, San Luis Potosi, SLP, Mexico; ^2^Escuela Superior de Odontología y Doctorado en Ciencias Biomédicas, Universidad Autónoma de Guerrero, Acapulco, GRO, Mexico

## Abstract

*Case Report*. A nine-year-old patient with a diagnosis of tuberous sclerosis (with no pathological record) that showed calcifications at the brain level. Besides, the case showed the Vogt triad (epilepsy, mental retardation, and sebaceous adenoma). The patient clinically showed a volume increase of hard consistency, without suppuration and no sessile that included the following teeth 73, 74, and 75. Cone beam computed tomography (CBCT) was obtained, and it displayed a delimited unilocular lesion. After surgical excision, the histopathological report was desmoplastic fibroma (DF). It was observed that the patient had an aggressive recurrence of DF at four months after surgery treatment. Due to these clinical findings, resective osseous surgery and curettage were carried out. It is uncommon to find these two pathologies together (DF and tuberous sclerosis). Since DF is a benign pathology but very invasive and destructive, it is necessary a constant follow-up examination due to a high recurrence frequency.

## 1. Introduction

Tuberous sclerosis (TS) is a rare disease described by Von Recklinghausen of neurocutaneous autosomal dominant origin, which is characterized by the development of benign tumors. This disease exhibits a triad known as the Vogt triad, the full triad that includes seizures, mental retardation, and cutaneous angiofibroma occuring in only 30% of reported cases. It is described by the presence of hamartomatous benign tumors, neurofibromas, and angiofibroma, which can be developed in the eyes, kidneys, heart, skin, brain, lungs, and progressive intracranial calcifications. There is a family history of the disease in 50% of all affected subjects, and the birth prevalence is as high as 1 in 6000 birth cases [1–3].

Oral manifestations of TS occur in 11% of affected subjects, and oral abnormalities include the presence of delayed eruption, bifid uvula, enamel hypoplasia, cystic hyperostosis, hyperplasia, enamel pitting, haemangioma, multiple osteomas, cleft lip and palate, and desmoplastic fibromas (DF) [2, 4]. The combination of desmoplastic fibroma and tuberous sclerosis is uncommon; there is little information about its clinical features. However, 84% of cases appear between the third and fourth decades of life, involving the mandible and maxillary area, having a predilection of 86% for the mandible and 14% in the maxilla. This tumor has a high percentage of relapse after surgical removal that usually occurs in 20%–40% of cases in which enucleation or excision of the fibroma occurs; while in cases with curettage, the relapse has a recurrence of 70%. Radiographically, a radiolucent area of a trabecular shape is seen, which is linked to soft tissue. The treatment is carried out using surgery, pharmacotherapy, or radiotherapy [5–10].

## 2. Case Presentation

**Figure 1:**
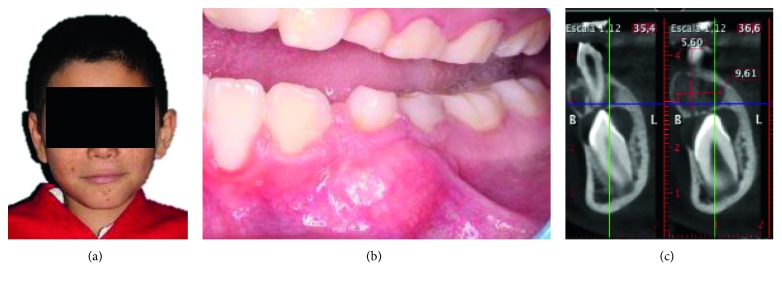
(a) Patient with sebaceous adenoma. (b) Oral examination revealed a facial asymmetry with a nonmovable mass about 8 mm in diameter 873 (74 and 75). (c) The tumor size is measured in the tomography, which is 5.6 × 9.6 × 8.3 mm in the left mandible site.

A nine-year-old patient with tuberous sclerosis, who was diagnosed at five months of age, presented the Vogt triad (sebaceous adenomas, epilepsy, and mental retardation). There was not a family history of tuberous sclerosis, epilepsy, nor mental retardation. However, cone beam computed tomography (CBCT) showed multiple radioopacities throughout the brain, which were diagnosed by the neuropediatrician (Figure 1(a)).

In March (2016), the patient attended to the dental clinic at San Luis Potosi University, oral examination revealed a facial asymmetry, and this increase of volume has no movement with a measure of about 8 mm in diameter overlying the left mandible from the canine to the second molar of the primary dentition. In addition, the enamel of permanent anterior teeth showed pitting or irregularities. Radiographs (panoramic and periapical) and a cone beam computed tomography (CBCT, Software Planmeca Romexis Viewer, Finland) were taken. The tomography shows the presence of a lesion of approximately 5.6 × 9.6 × 8.3 mm, and the cortical bone was covered with an incomplete fibrous capsule. Radiographic examination of the mandible revealed a large, round, radiolucent lesion with edges well circumscribed (Figures 1(b) and 1(c)). Therefore, it was decided to remove the lesion and perform a histopathological study.

## 3. Surgical Procedure

**Figure 2:**
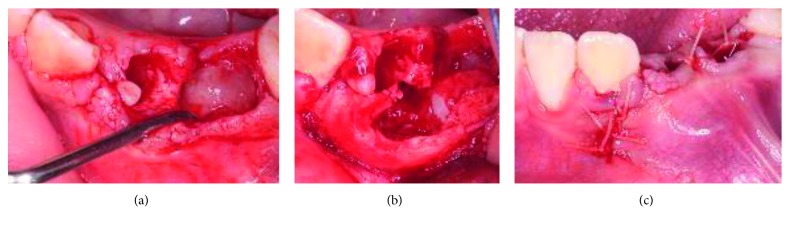
(a) The deciduous teeth 73 and 74 were removed. (b) A surgical enucleation was carried out. (c) The flap was repositioned and sutured with Vicryl 4-0.

Before the clinical procedure, the caregivers received an informed consent form where ethical principles were taken into consideration based on the Declaration of Helsinki on Ethical Principles for Medical Research Involving Human Subjects (version 2013). After the acceptance of the treatment by their parents, asepsis with iodopovidone was performed in the patient in the intervention site; local infiltration of lidocaine containing 2% of epinephrine (Zeyco FD, Mexico) was carried out. Deciduous teeth 73 and 74 were removed, and then a full-thickness flap technique was performed; debridement and surgical enucleation of the lesion (10 × 15 × 13 mm) were carried out, ensuring that no fibrous tissue was left in the area. Subsequently, the flap was repositioned and sutured with Vicryl 4-0 (Ethicon, Polyglactin 910, USA). The biopsy was sent to the pathology department, and the following week, the patient attended to his follow-up appointment for stitches removal (Figures 2(a)–2(c)).

**Figure 3:**
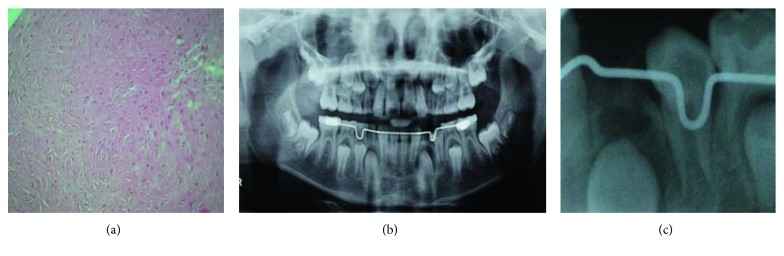
(a) The histopathological result was desmoplastic fibroma, which was constituted by a proliferation of connective tissue with the presence of fusiform fibroblasts. (b) Radiographic control after first surgery. (c) At the fourth month follow-up, it was observed in the radiograph a radiolucent area apically around the left mandibular canine (33).

The histopathological showed a DF, constituted by a proliferation of connective tissue with the presence of fusiform fibroblasts deposited between dense hyalinized collagen bundles (Figure 3(a)). Once the diagnosis was established, the patient was referred for monthly follow-ups (clinical and radiographic evaluation) to assess the eruption of permanent teeth (33 and 34). Also, the patient was referred to the pediatric dentist for placement of a space maintainer.

In the clinical follow-ups, radiographically was observed that the first premolar (34) during the first three months, and there was no visible lesion (Figure 3(b)). However, in fourth-month control appointment, the presence of tissue adjacent to the erupting teeth was observed radiographically, as well as an asymmetry in the left mandible, so a second intervention was decided (Figure 3(c)). An asepsis technique was performed with iodopovidone and infiltration with lidocaine 2% with epinephrine (Zeyco FD, Mexico). A full-thickness flap technique was made for accessing to the lesion by the vestibular site, especially to have a clean access for the left-lower permanent canine. During the surgery procedure, multiple fragments of both soft and hard tissues were removed; the fragments were sent to the pathology department with a diagnosis of a presumption of recurrent desmoplastic fibroma.

**Figure 4:**
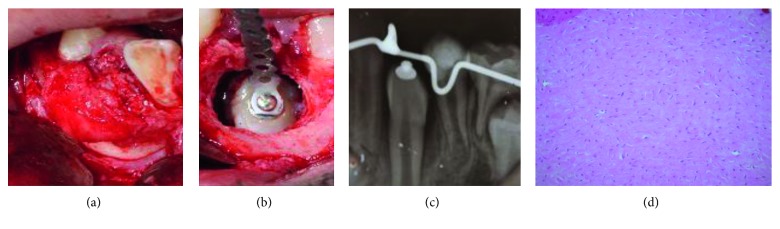
(a) At the fourth month, a surgical excision was performed, and the lesion was observed delimited and adhered to adjacent tissues. (b) The lesion was removed, and an osteotomy was performed. (c) A button was placed on the left mandibular canine, and a close orthodontic chain without traction was placed. (d) Histopathological result of 4 months of desmoplastic fibroma relapse.

**Figure 5:**
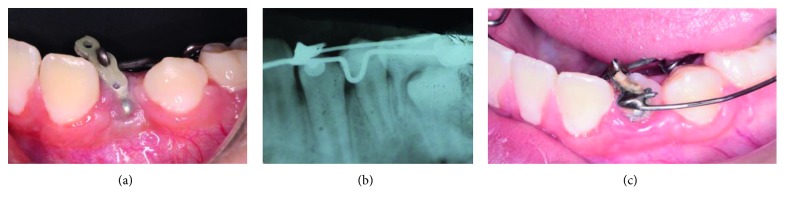
(a) Two months after the last surgery. (b) The eruption of canine. (c) Five months follow-up.

An osseous resective surgery with safe margins and curettage of the surgical site was performed with a subsequent use of the orthodontic button onto the left mandible canine. It was placed a closed chain without traction (Figure 4(a)). Finally, the flap was repositioned by using Vicryl Suture 4-0 (Ethicon, Polyglactin 910, USA), leaving the orthodontic chain free (Figure 4(b)). Eight days later, the patient had an appointment for suture removal and then referred to his pediatric dentist for the space maintainer placement. The Department of Oral Pathology reported the presence of desmoplastic fibroma recurrence with bundles of spindle-shaped, fibroblast-like cells in a collagen matrix (Figure 5(a)). The canine was erupted after 5 months (Figures 5(b) and 5(c)).

## 4. Discussion

There is little information about the combination of desmoplastic fibroma and tuberous sclerosis; this combination is uncommon [8, 10, 11]. Likewise, there are no epidemiologic studies due to its low frequency, and the features are not well-known. In the world, there are few cases reported; however, some authors described a higher prevalence of desmoplastic fibroma in the mandible compared to the maxilla, as occurred in this case report [8, 12, 13].

It has been observed radiographically that relapsing lesions are unilocular or multilocular; the patient showed a unilocular radiolucent area at four months of the first surgery, coronal to the erupting dental organ 33 [5, 11]. DF is a collagenous lesion that lacks odontogenic epithelium; for maxillofacial surgeons, this information is crucial since a wider margin of resection is required when provided definitive treatment and probably it could be the cause of relapsing lesions. There is a controversy related to the presence of pain; the patient did not show pain prior the surgical treatment, despite the aggressive growth of the lesion. This clinical finding is in accordance with other reports; however, there are reports of pain due to the growth of the lesion [8, 14]. The removed DF showed lower size than previous reports, where the lesions had variable sizes of 2 to 7 cm [7, 14, 15].

Establishing a diagnosis of DF is difficult by imaging studies alone, since many tumors resemble DF. The differential diagnosis includes a wide range of lesion from benign lesions (fibrous dysplasia, bone cysts ossifying fibroma, and eosinophilic granuloma) to malignant lesions (osteosarcoma). In addition, DF includes pathologies from soft tissues (desmoplastic fibroma) to bone tissues (bone desmoid tumor). Besides, due to its high recurrence, osseus resective surgery with safe margins and curettage should be considered as the optimal treatment. The follow-up examination is an important issue to detect recurrence; it should be done monthly for the first year.

## 5. Conclusion

In the present case report, it was observed that the patient had an aggressive recurrence of DF at four months after the first surgical resection; it was necessary a second surgery that included safe margins and curettage. However, most reported cases did not mention the relapse time, just a high rate of recurrence. Besides, it is uncommon to find these two pathologies together (DF and tuberous sclerosis). Furthermore, it is necessary an excellent follow-up control due to a high recurrence frequency. The DF is benign pathology but is very invasive and destructive.
